# Hard nut to crack: Solving the disulfide linkage pattern of the *Neosartorya* (*Aspergillus*) *fischeri* antifungal protein 2

**DOI:** 10.1002/pro.4692

**Published:** 2023-07-01

**Authors:** Györgyi Váradi, Zoltán Kele, András Czajlik, Attila Borics, Gábor Bende, Csaba Papp, Gábor Rákhely, Gábor K. Tóth, Gyula Batta, László Galgóczy

**Affiliations:** ^1^ Department of Medical Chemistry Albert Szent‐Györgyi Medical School, University of Szeged Szeged Hungary; ^2^ Department of Organic Chemistry, Faculty of Science and Technology University of Debrecen Debrecen Hungary; ^3^ Department of Biochemistry Institute of Biochemistry and Molecular Biology, Semmelweis University Budapest Hungary; ^4^ Laboratory of Chemical Biology Institute of Biochemistry, Biological Research Centre, Eötvös Loránd Research Network Szeged Hungary; ^5^ Department of Biotechnology Faculty of Science and Informatics, University of Szeged Szeged Hungary; ^6^ Department of Microbiology Faculty of Science and Informatics, University of Szeged Szeged Hungary; ^7^ Institute of Biophysics, Biological Research Centre, Eötvös Loránd Research Network Szeged Hungary; ^8^ MTA‐SZTE Biomimetic Systems Research Group, University of Szeged Szeged Hungary; ^9^ Fungal Genomics and Evolution Lab, Institute of Biochemistry, Biological Research Centre, Eötvös Loránd Research Network Szeged Hungary

**Keywords:** antifungal protein, disulfide linkage pattern, drug design, protein structure, protein synthesis

## Abstract

As a consequence of the fast resistance spreading, a limited number of drugs are available to treat fungal infections. Therefore, there is an urgent need to develop new antifungal treatment strategies. The features of a disulfide bond‐stabilized antifungal protein, NFAP2 secreted by the mold *Neosartorya* (*Aspergillus*) *fischeri* render it to be a promising template for future protein‐based antifungal drug design, which requires knowledge about the native disulfide linkage pattern as it is one of the prerequisites for biological activity. However, in the lack of tryptic and chymotryptic proteolytic sites in the ACNCPNNCK sequence, the determination of the disulfide linkage pattern of NFAP2 is not easy with traditional mass spectrometry‐based methods. According to *in silico* predictions working with a preliminary nuclear magnetic resonance (NMR) solution structure, two disulfide isomers of NFAP2 (*abbacc* and *abbcac*) were possible. Both were chemically synthesized; and comparative reversed‐phase high‐performance liquid chromatography, electronic circular dichroism and NMR spectroscopy analyses, and antifungal susceptibility and efficacy tests indicated that the *abbcac* is the native pattern. This knowledge allowed rational modification of NAFP2 to improve the antifungal efficacy and spectrum through the modulation of the evolutionarily conserved γ‐core region, which is responsible for the activity of several antimicrobial peptides. Disruption of the steric structure of NFAP2 upon γ‐core modification led to the conclusions that this motif may affect the formation of the biologically active three‐dimensional structure, and that the γ‐core modulation is not an efficient tool to improve the antifungal efficacy or to change the antifungal spectrum of NFAP2.

## 1 INTRODUCTION

As a consequence of the emerging number of life‐threatening fungal infections caused by antifungal‐drug resistant strains, there is an urgent need to develop new treatment strategies applying antifungal compounds that differ from the existing ones in chemical features and mechanism of action (Kainz et al., 2020). Besides the novel chemical therapeutics targeting fungal cell wall, cell membrane, and intracellular targets (Rauseo et al., 2020), natural and synthetic antifungal peptides (Fernández de Ullivarri et al., 2020) and proteins (AFPs) represent alternative drug candidates; among them, the *Neosartorya* (*Aspergillus*) *fischeri* antifungal protein 2 (NFAP2) of filamentous fungal origin (Galgóczy et al., 2019). NFAP2 inhibits the growth of opportunistic human pathogen *Candida* species and eradicates their drug‐resistant biofilms alone or in synergistic combination with licensed antifungal drugs (Kovács et al., 2021; Tóth et al., 2018). The experimentally determined efficacy of NAFP2 in a murine vulvovaginal candidiasis model (Kovács et al., 2019), and a three‐dimensional human skin model (Holzknecht et al., 2022) already support its therapeutic potential in the safe treatment of (antifungal drug‐resistant) superficial fungal infections. Taking these features into account, NFAP2 is considered a promising template for future protein‐based antifungal drug design (Galgóczy et al., 2019).

Similar to other AFPs of filamentous fungal origin, NFAP2 is a small molecular weight, highly stable, cationic, cysteine‐rich extracellular protein (Table 1) (Galgóczy et al., 2019). Its β‐pleated conformation is stabilized by three intramolecular disulfide bonds between six cysteine residues (Tóth et al., 2016). This last feature is essential for the proper folding and structural stability of several AFPs (Batta et al., 2009; Galgóczy et al., 2017; Lacadena et al., 1995; Váradi et al., 2013). Bulk production of recombinant NFAP2 (rNFAP2) in a *Penicillium chrysogenum*‐based expression system and a method for the chemical synthesis of properly folded and biologically active NFAP2 have already been developed (Tóth et al., 2018). The proper folding of the recombinant and the synthetic NFAP2 suggests the presence of native disulfide linkage pattern in both (Tóth et al., 2018). In living cells, well‐defined and regulated redox pathways catalyze the correct formation of disulfide bonding patterns, and enzymes degrade misfolded proteins (Sevier & Kaiser, 2002). On the contrary, the chemical synthesis allows arbitrary disulfide linkage pattern formation by application of orthogonal protection for cysteine thiols to generate different disulfide isomers of a protein including the native one (Váradi et al., 2013; Váradi et al., 2018). In most cases, the protection of the side chains of cysteines during the synthesis is not necessary to form the natural disulfide bonding pattern as it was observed at NFAP2, where a glutathione redox buffer is appropriate and enough for correct folding (Tóth et al., 2018; Váradi et al., 2018). However, the natural disulfide linkage pattern of NFAP2 is still unknown; because the determination of disulfide connectivity in NFAP2 with traditional mass spectrometry‐based methods is not easy in the lack of tryptic and chymotryptic proteolytic sites in the ACNCPNNCK sequence of the primary protein structure (Figure S1). One solution to overcome this problem is the application of disulfide linkage pattern prediction servers and software.

**TABLE 1:** Amino acid sequences and in silico predicted physicochemical properties of the investigated *Neosartorya* (*Aspergillus*) *fischeri* antifungal protein 2 (NFAP2) and the γ‐core modified variant (NFAP2γ).

Protein/peptide	Number of amino acids^a^	Molecular weight (kDa)^a^	Number of Cys^a^	Number of Lys/Arg/His^a^	Theoretical pI^a^	Estimated charge at pH 7^b^	GRAVY^a^
IATSPYYACNCPNNCKHKKGSGCKYHSGPSDKSKVIS **GKCEWQGGQLNC** IAT							
**NFAP2**	52	5.6	6	7/0/2	9.02	+5.2	−0.731
γ‐core	12	1.3	2	1/0/0	5.99	−0.2	−0.933
IATSPYYACNCPNNCKHKKGSGCKYHSGPSDKSKVIS **GKCKTKKNKC** IAT							
**NFAP2γ**	50	5.4	6	11/0/2	9.36	+10.2	−0.918
γ‐core	10	1.1	2	5/0/0	9.90	+4.8	−1.910

*Note*: The γ‐core motif is indicated with underlined letters in the primary structure.

Abbreviation: GRAVY, grand average of hydropathy.

^a^ ExPASy ProtParam tool (Gasteiger et al., 2005).

^b^ Protein Calculator v3.4 server (The Scripps Research Institute; http://protcalc.sourceforge.net/).

NFAP2 is an extracellular protein; therefore, all cysteine residues should be oxidized and form disulfide bridges. Considering the presence of six cysteines, 15 different disulfide bridge patterns are possible. Available disulfide connectivity prediction servers operating with the primary structure of NFAP2 indicated three probable and different bonding patterns: *abcabc* (DISULFIND) (Ceroni et al., 2006), *aabcbc* (DIANNA) (Ferrè & Clote, 2006), and *abbacc* (Savojardo et al., 2011). The solution structure of cysteine‐rich AFPs could be well‐characterized by nuclear magnetic resonance (NMR) spectroscopy (Batta et al., 2009; Czajlik et al., 2021; Fizil et al., 2015; Fizil et al., 2018; Hajdu et al., 2019; Huber et al., 2018), although the disulfide bridges are invisible in this method (Wiedemann et al., 2020). As a solution for this problem, computational approaches operating with preliminary three‐dimensional solution structures have been developed to predict disulfide bridge formation and bonding patterns of cysteine‐rich proteins. Applying a preliminary NMR structure of NFAP2 (Supporting Information), the Disulfide by Design 2.0 server (Craig & Dombkowski, 2013) predicted disulfide bridges between Cys9‐Cys40, and Cys23‐Cys49, and indicated no bond between Cys11 and Cys15. However, a disulfide bond between these last two cysteine residues is possible under oxidative conditions considering that they are located in a highly flexible loop region (Tóth et al., 2018) (Figure S2), resulting in *abbcac* pattern. It is not evident which one of the above‐mentioned prediction methods represents reality because the results and accuracy of these computational approaches highly depend on several factors, for example, the data set used, and the input features of the machine learning algorithm. Therefore, the results of the prediction on the disulfide connectivity can be misleading (Márquez‐Chamorro & Aguilar‐Ruiz, 2015).

Several biomolecules are considered potential new drug candidates or represent potential templates for future drug design. This latter goal requires knowledge about the biologically active structure, and the structural features responsible for the activity. The correct native disulfide linkage pattern is one of the prerequisites for the biological activity of therapeutic proteins (Weinfurtner, 2018). Its determination in NFAP2 is critically important to understanding the structure–function relationships. A structural motif of AFPs, which can be used for antifungal protein‐based drug design, is the evolutionarily conserved γ‐core region. It is usually located in a loop region and constituted by the consensus amino acid sequence GXC‐X_3‐9_‐C, where X can be any amino acid (in NFAP2: GKCEWQGGQLNC) (Table 1) (Sonderegger et al., 2018). It has already been evidenced that modulation of the γ‐core region (rational amino acid substitutions which increase the positive charge and hydrophilicity) can improve the antifungal efficacy (Sonderegger et al., 2018), or change the antifungal spectrum of an AFP, such as the *Penicillium chrysogenum* antifungal protein, PAF (Tóth et al., 2020). Furthermore, rationally designed synthetic peptides spanning the native or modified γ‐core inhibit the fungal growth alone (Sonderegger et al., 2018; Tóth et al., 2020).

Taking into account the above‐mentioned importance of the knowledge about the native disulfide bonding pattern of NFAP2, and that this protein can be considered a promising template for future drug design, the present study aimed to determine the disulfide connectivity experimentally. Since this aim could not be achieved easily using traditional mass spectrometry‐based methods in the lack of tryptic and chymotryptic proteolytic sites in the ACNCPNNCK sequence (Figure S1), the most probable disulfide isomers of NFAP2 predicted by computational methods were chemically synthesized, and their structures and antifungal efficacy were analyzed and compared with that of the native protein. In the hand of the native disulfide bonding pattern, the impact of the γ‐core motif modulation on the antifungal efficacy and structure of NFAP2 was also studied.

## 2 RESULTS

### 2.1 Chemical synthesis of NFAP2 disulfide isomers

**FIGURE 1:**
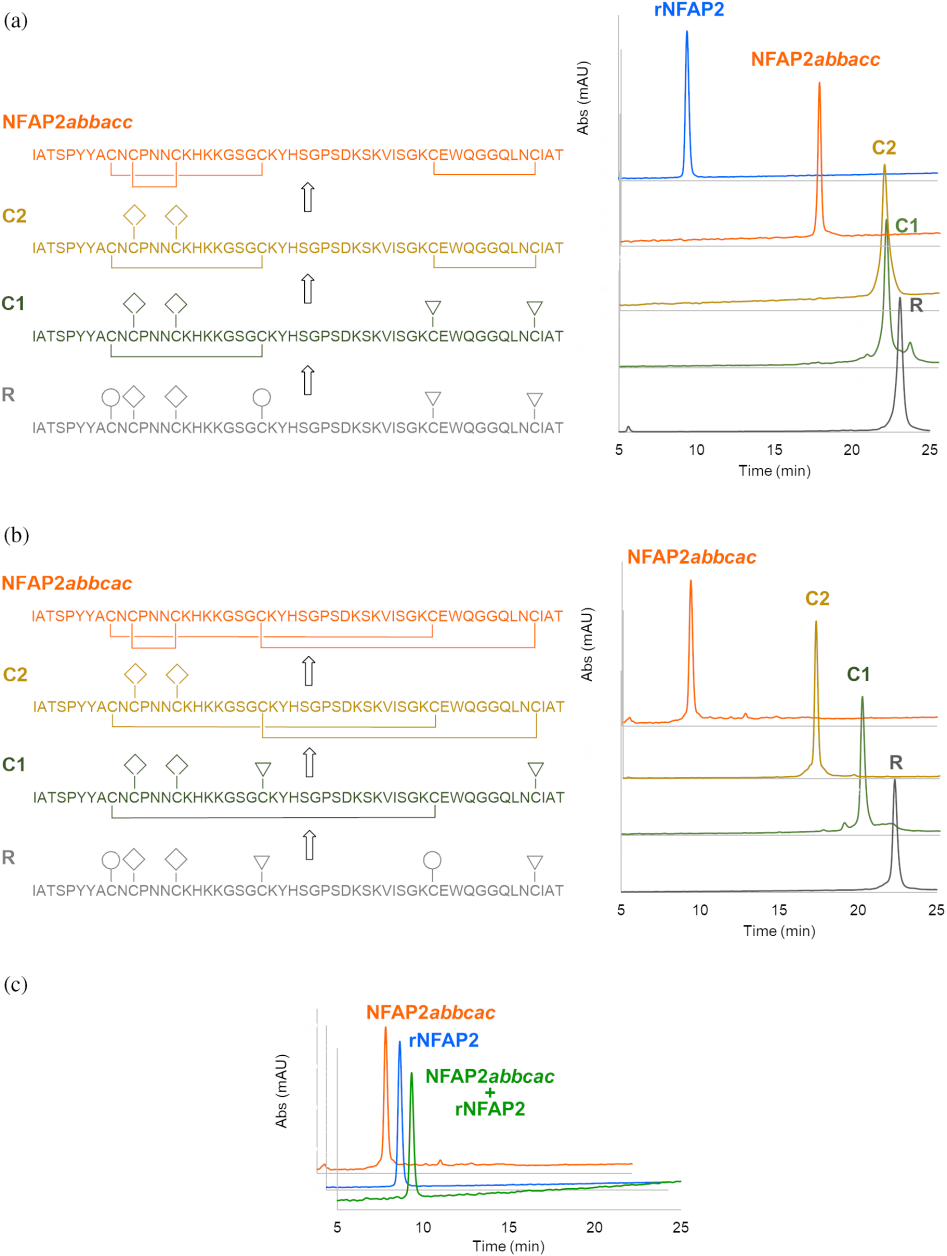
Cysteine pairing and RP‐HPLC elution profiles of intermediates in stepwise disulfide bond formation of *Neosartorya* (*Aspergillus*) *fischeri* antifungal protein 2 (NFAP2) disulfide isomers (a) NFAP2*abbacc* and (b) NFAP2*abbcac*. Protecting groups are labeled as follows: ◯, Trt; ▽, Acm; ◇, Mob. The chromatogram of recombinant NFAP2 (rNFAP2) possessing the native disulfide linkage pattern is shown in panel (a) for comparison. (c) The co‐elution profile of NFAP2*abbcac* and rNFAP2 reveals the identity of the two proteins.

**FIGURE 2:**
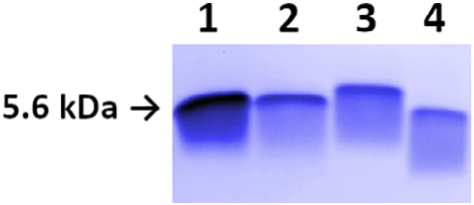
Electrophoretic mobility of recombinant *Neosartorya* (*Aspergillus*) *fischeri* antifungal protein 2 (rNFAP2), its disulfide isomers (NFAP2*abbcac*, NFAP2*abbacc*) and γ‐core variant (NFAP2γ) in 18% (w/v) in tris‐glycine SDS‐PAGE. Lane 1: rNFAP2 (5.6 kDa; 4 μg), lane 2: NFAP2*abbcac* (2 μg), lane 3: NFAP2γ (2 μg), lane 4: NFAP2*abbacc* (2 μg). The same electrophoretic mobility of NFAP2*abbcac* and rNFAP2 reveals the structural identity of the two proteins; whereas the different mobility of NFAP2γ and NFAP2*abbacc* indicates their different structure than that of rNFAP2 and NFAP2*abbcac*.

To decide which predicted disulfide isomers of NFAP2 are close to reality and worth being synthesized, the distance between cysteine pairs in the preliminary tertiary structure (Figure S2) was taken into consideration. A distance criterion between Cα atoms of a cysteine pair in the range of 3.0 Å and 7.5 Å was used (Gao et al., 2020). Disulfide isomers with a distance out of this range at least on one cysteine pair (*abcabc* and *aabcbc*) were excluded (Figure S2), and those where all cysteine pairs met the criterion, namely the *abbacc* and the *abbcac* isomers (Figure S2), were synthesized. The application of orthogonal thiol protecting groups ensured the regioselective formation of the three disulfide bonds. The process was followed by reversed‐phase high‐performance liquid chromatography (RP‐HPLC). Figure 1a,b shows the consecutive intermediates of the synthesis of the NFAP2*abbacc* and the NFAP2*abbcac* disulfide isomers, respectively. RP‐HPLC analysis revealed the identity of NFAP2*abbcac* and rNFAP2 heterologously produced in *P. chrysogenum* (Figure 1c). The retention times of NFAP2 intermediates gradually decreased in parallel with the increase in the number of disulfide bridges. This observation suggests that the formation of disulfide bridges induces more compact, folded structures (Figure 1a,b). NFAP2*abbcac* showed the same electrophoretic mobility as rNFAP2 in tris‐glycine 18% (w/v) sodium dodecyl sulfate‐polyacrylamide gel electrophoresis (SDS‐PAGE), while NFAP2*abbacc* appeared below the rNFAP2 band further proving the structural difference (Figure 2). Figures S3 and S4 show the mass spectrum of NFAP2*abbacc* and NFAP2*abbcac*, respectively.

### 2.2 Structural investigations of NFAP2 disulfide isomers

**FIGURE 3:**
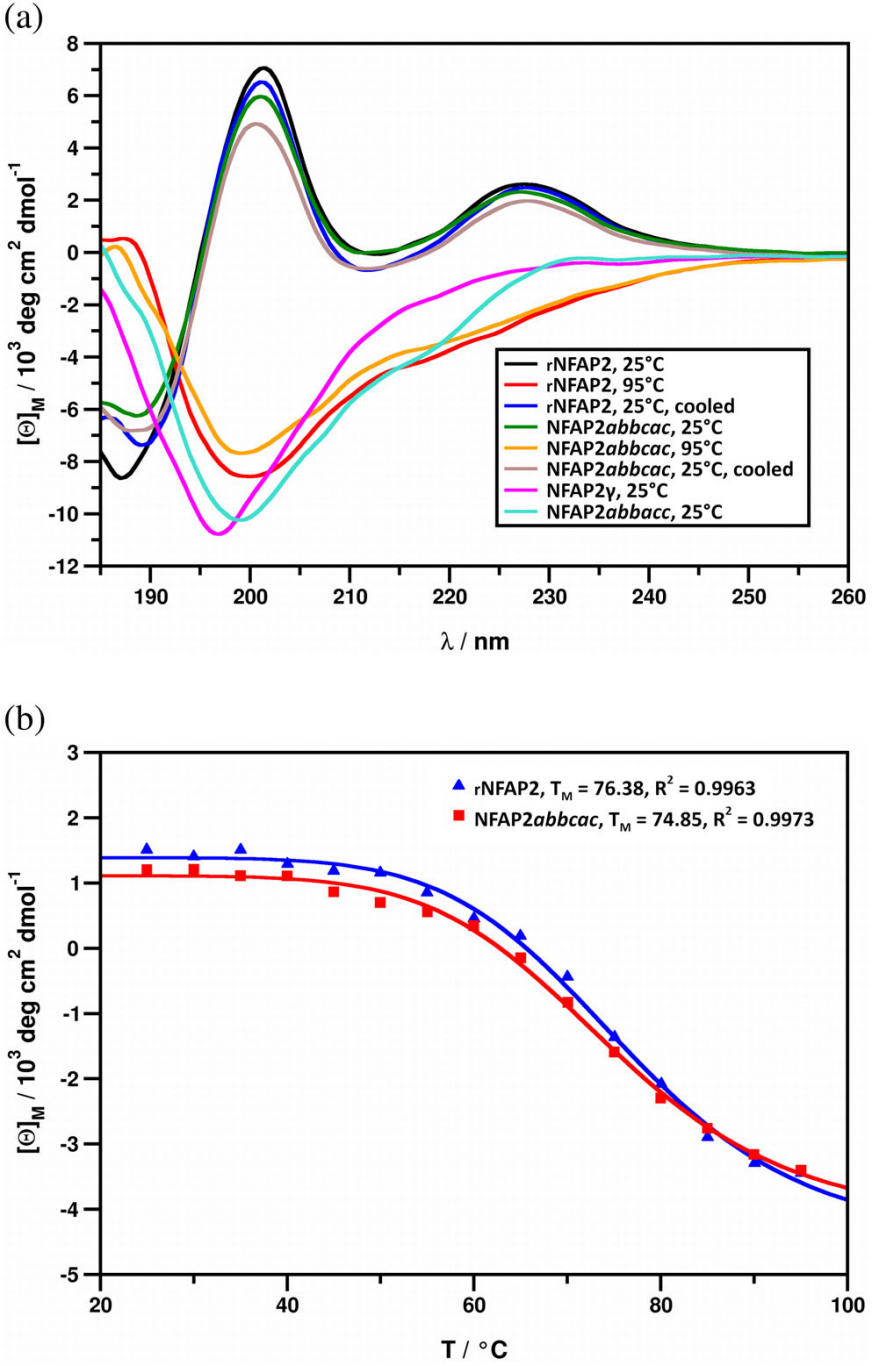
(a) ECD spectra of the recombinant *Neosartorya* (*Aspergillus*) *fischeri* antifungal protein 2 (rNFAP2), its disulfide isomers (NFAP2*abbcac*, NFAP2*abbacc*), and γ‐core variant (NFAP2γ) at different temperatures: rNFAP2 at 25°C (black), rNFAP2 at 95°C (red), rNFAP2 cooled back to 25°C after annealing (blue), NFAP2*abbcac* at 25°C (green), NFAP2*abbcac* at 95°C (orange), NFAP2*abbcac* cooled back to 25°C after annealing (light brown), NFAP2*abbacc* at 25°C (cyan), NFAP2γ at 25°C (magenta). (b) Thermal unfolding curves of rNFAP2 (blue, *T*_*m*_ = 76.38°C, *R* ^2^ = 0.9963) and NFAP2*abbcac* (red, *T*_*m*_ = 74.85°C, *R* ^2^ = 0.9973) measured at 228 nm.

**FIGURE 4:**
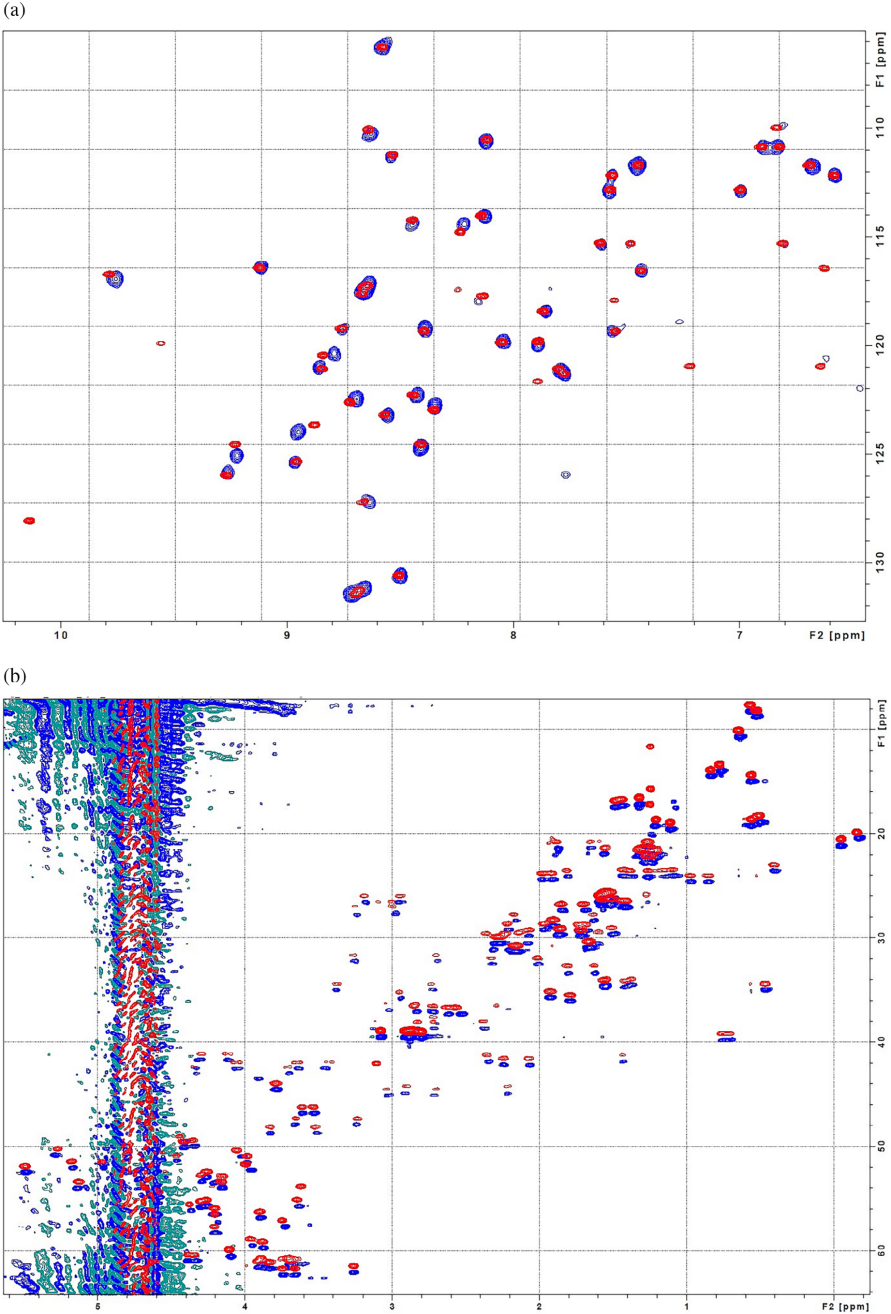
Comparison of the NMR spectra of the recombinant *Neosartorya* (*Aspergillus*) *fischeri* antifungal protein 2 (rNFAP2, red) and the synthetic NFAP2*abbcac* disulfide isomer (blue). (a) Overlaid ^15^N‐HSQC spectra. (b) Overlaid ^13^C‐HSQC spectra. The spectra are intentionally shifted for better comparison. The spectral identity indicates the same protein structure of rNFAP2 and NFAP2*abbcac*.

The native disulfide bonding pattern suggested by RP‐HPLC retention times was corroborated by electronic circular dichroism (ECD) spectra of the rNFAP2 possessing the native pattern and NFAP2 synthetic disulfide isomers. Whereas the spectra of rNFAP2 and that of NFAP2*abbcac* were identical, demonstrating the same features as spectra measured previously for AFPS of filamentous fungal origin (Fizil et al., 2015; Galgóczy et al., 2017; Garrigues et al., 2017; Tóth et al., 2018), the spectrum acquired for NFAP2*abbacc* indicated unordered structure (Figure 3a). The structures of rNFAP2 and NFAP2*abbcac* responded similarly to elevated temperature, and the structural destabilization and unfolding were reversible for both proteins. Loss of the native folded structure was observed in measurements executed at 95.0°C, but the native structure was restored after samples of these proteins were allowed to cool back to ambient temperatures. The thermal unfolding curves of these two proteins, recorded with ECD detection were also nearly identical (Figure 3b). The difference between the curves was within the margin of experimental error and the corresponding melting temperatures of protein structures (*T*_*m*_) were calculated as 76.38°C and 74.85°C, respectively. These results indicate that the rNFAP2 has the same disulfide linkage pattern as the NFAP2*abbcac*, therefore the *abbcac* is the native one. The difference between the spectra of rNFAP2 and NFAP2*abbcac* measured at 95.0°C compared to that of NFAP2*abbacc* suggests that the structure of this latter disulfide isomer is not partially folded, but completely misfolded with regard to the native structure. NMR spectroscopy further evidenced the ECD results. As can be seen in Figure 4, the ^15^N and ^13^C NMR spectra of NFAP2*abbcac* and rNFAP2 show nearly perfect overlap for visual inspection. Since chemical shifts are sensitive indicators of folded protein conformations (e.g., structures can be predicted from chemical shifts [ROSETTA, etc.]), the good agreement between NFAP2*abbcac* and rNFAP2 NMR spectra proves similar conformations, that can happen only with identical disulfide bonding patterns.

### 2.3 Chemical synthesis and structural investigation of an NFAP2 variant possessing modified γ‐core

**FIGURE 5:**
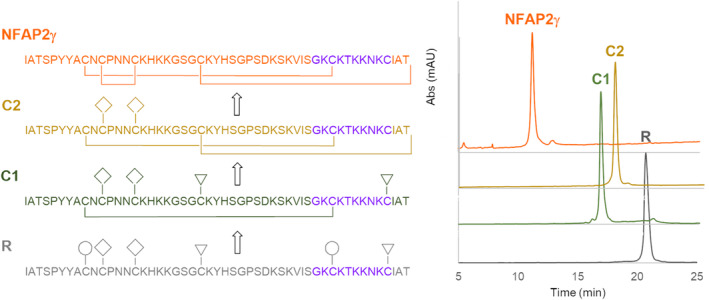
Cysteine pairing and RP‐HPLC elution profiles of intermediates in stepwise disulfide bond formation of a γ‐core variant of the *Neosartorya* (*Aspergillus*) *fischeri* antifungal protein 2 (NFAP2γ). The modified γ‐core motif is highlighted in purple in the sequence. Protecting groups are labeled as follows: ◯, Trt; ▽, Acm; ◇, Mob.

RP‐HPLC analysis, ECD, ^15^N and ^13^C NMR spectroscopy investigations revealed the identity of NFAP2*abbcac* and rNFAP2 proteins. Solving the puzzle of disulfide bond patterns opened the door for the design and synthesis of NFAP2 variants for the investigation of structure–function relationships. The evolutionary conserved γ‐core motif, which is known to affect either the efficacy and/or the structure of antimicrobial peptides and proteins (Sonderegger et al., 2018; Yeaman & Yount, 2007; Yount & Yeaman, 2004), was targeted by these modifications. In our previous study, we already proved that a rationally designed peptide variant spanning the NFAP2 γ‐core region (Table 1) with increased positive charge and hydrophilicity (Ac‐VISGKC(‐SH)KTKKNKC(‐SH)K‐NH_2_) showed antifungal activity against several plant pathogenic filamentous fungi (Tóth et al., 2022). These results led us to synthesize the γ‐core variant of NFAP2 harboring this rationally designed γ‐core motif (NFAP2γ) (Table 1) and examine it in comparison with the rNFAP2 and synthetic NFAP2 possessing *abbcac* disulfide connectivity. Regioselective formation of disulfide bonds (*abbcac*) was performed by applying the same set of sulfhydryl‐protecting groups as for the previously mentioned NFAP2 disulfide isomers. RP‐HPLC analysis showed a very rare shift in the retention times of the intermediates, namely, C1 (Cys9‐Cys23 or Cys9‐Cys40) eluted at a shorter retention time than C2 (Cys9‐Cys23, Cys40‐Cys19 or Cys9‐Cys40, Cys23‐Cys49) (Figure 5). Figure S5 shows the mass spectrum of NFAP2γ. In tris‐glycine 18% (w/v) SDS‐PAGE, NFAP2γ appeared above the rNFAP2 band (Figure 2). ECD spectroscopic analysis of this protein revealed similar features to that of rNFAP2 and NFAP2*abbcac* acquired at 95.0°C, indicating a very loosely folded, dominantly unordered structure (Figure 3).

### 2.4 Antifungal efficacy of NFAP2 disulfide isomers and γ‐core variant

The proper tertiary structure stabilized by the correct native disulfide linkage pattern is essential for the biological activity of therapeutic proteins (Weinfurtner, 2018); therefore, we investigated the antifungal efficacy of NFAP2 disulfide isomers and its γ‐core variant. NFAP2*abbcac* showed the same minimum inhibitory concentration (MIC) (6.25 μg mL^−1^) against the human pathogenic yeast *Candida albicans* CBS 5982 in the applied *in vitro* susceptibility test as that of rNFAP2. In contrast, NFAP2γ and NFAP2*abbacc* possessed higher MICs (MIC = 12.5 μg mL^−1^ and 50 μg mL^−1^, respectively). Propidium iodide (PI) is a membrane‐impermeable, red‐fluorescent nuclear and chromosome stain. It stains *Candida* cells red if cell membranes are disrupted, which indicates cell death. Therefore, PI‐coupled fluorescence‐activated cell sorting (FACS) analysis is an appropriate method to monitor the number of dead cells in the presence of NFAP2, its disulfide isomers, and γ‐core variant and to deduce their antifungal efficacy. FACS analysis indicated that there was a significant difference between the *C. albicans* cell‐killing efficacies of the studied NFAP2 disulfide isomers (*F*_3,4_ = 8.4672, *p* < 0.05). However, a notable difference was not observed between the proportion of dead cells when they were treated with rNFAP2 (27.6 ± 0.57%), NFAP2*abbcac* (32.7 ± 2.69%), or NFAP2γ (30.1 ± 3.46%), but significant difference existed between the treatments with NFAP2*abbcac* and NFAP2*abbacc* (21.9 ± 0.71%) (*p* < 0.05 according to Tukey's HSD post‐hoc test). All these results indicated that the disulfide bond pattern and the amino acid composition of the γ‐core motif influence the antifungal efficacy of NFAP2. These data are summarized in Table 2.

**TABLE 2:** Minimum inhibitory concentration (MIC) and cell killing efficacy of recombinant *Neosartorya* (*Aspergillus*) *fischeri* antifungal protein 2 (rNFAP2), its disulfide isomers (NFAP2*abbcac*, NFAP2*abbcac*), and γ‐core variant (NFAP2γ) against *Candida albicans* CBS 5982.

NFAP2 variant	MIC (μg mL^−1^)	Cell killing efficacy (%)
rNFAP2	6.25	27.6 ± 0.57%
NFAP2*abbcac*	6.25	32.7 ± 2.69%
NFAP2*abbacc*	50	21.9 ± 0.71%*
NFAP2γ	12.5	30.1 ± 3.46%

*Note*: MIC was defined as the lowest protein concentration at which growth was ≤5% in comparison with the untreated control. Cell killing efficacy was determined after treatment for 16 h at 30°C under shaking at 160 rpm.

* Significant difference between the treatments with NFAP2*abbcac* and NFAP2*abbacc* (*p* < 0.05).

## 3 DISCUSSION

Despite the introduction of more and more sophisticated methods, accurate prediction of disulfide bonding patterns in cysteine‐rich proteins is still challenging (Márquez‐Chamorro & Aguilar‐Ruiz, 2015). In most cases, an experimental determination is essential to confirm the *in silico* results and to understand the impact of the disulfide bonding pattern on the structural‐functional relationships, especially, when a protein is considered as a potential template for future drug design (Tang & Speicher, 2019), such as fungal AFPs (Galgóczy et al., 2019). Crystal diffraction data analyses, NMR assignments with MS‐coupled proteolytic digestion methods proved to be appropriate to determine and confirm the disulfide connectivity in different AFPs possessing six cysteine residues, such as *abcabc* in *P. chrysogenum* PAF (Batta et al., 2009), PAFB (Huber et al., 2018), and *N*. (*A*.) *fischeri* NFAP (Hajdu et al., 2019); and *abcdabcd* in *P. brevicompactum* BP (Olsen et al., 2004) and *P. chrysogenum* PAFC (Czajlik et al., 2021). However, these methods are not easily applicable for NFAP2 to determine the disulfide linkage pattern; because NFAP2 has a highly flexible N‐terminal loop region (Tóth et al., 2018), some residues are invisible in NMR due to molecular dynamics features, moreover, tryptic as chymotryptic proteolytic sites in the ACNCPNNCK sequence of the primary protein structure are not present (Figure S1). This problem could be overcome by the creation of different NFAP2 disulfide isomers with the application of orthogonal protection for the cysteine thiols, thus, enabling the regioselective formation of the three disulfide bridges. Since one of the disulfide isomers, NFAP2*abbcac* showed the same retention time (Figure 1b), ECD spectrum (Figure 3a), melting temperature (Figure 3b), NMR spectra (Figure 4), and antifungal efficacy as those of rNFAP2, this disulfide bonding pattern variant was proved to be the native one.

It has already been reported that the presence of all disulfide bridges and the formation of native disulfide bonding pattern are the prerequisites for the structural integrity and the full antifungal activity of different AFPs, such as *A. giganteus* AFP (Lacadena et al., 1995), *P. chrysogenum* PAF (Batta et al., 2009; Váradi et al., 2013), and *N. fischeri* NFAP (Galgóczy et al., 2017). This statement is also true for NFAP2 considering that the non‐native *abbacc* disulfide isomer showed an ECD spectrum that was characteristic of unordered proteins (Figure 3a) and possessed decreased antifungal efficacy in comparison with rNFAP2 and NFAP2*abbcac*.

The determination of the native *abbcac* disulfide bonding pattern paved the way for the rational modification of the NFAP2 structure to improve the antifungal efficacy or modulate the antifungal spectrum. One of the designable structural elements of AFPs, for this reason, is the evolutionarily conserved γ‐core motif. It is present in almost all cysteine‐rich extracellular antimicrobial peptides and proteins of living organisms and has important functional and structural roles (Yeaman & Yount, 2007; Yount & Yeaman, 2004). It was already demonstrated at antifungal active plants defensins (de Oliveira et al., 2019; Olsen et al., 2004; Sagaram et al., 2011), and at the *P. chrysogenum* PAF (Sonderegger et al., 2018) that rational substitutions of negatively charged and neutral amino acid residues with positively charged ones in the γ‐core region increase the antifungal efficacy. Our results with NFAP2 somehow contradicted this observation as the efficacy of NFAP2 did not decrease significantly when we exchanged the almost neutral and slightly hydrophilic γ‐core region for a rationally designed positively charged and more hydrophilic one (Tables 1 and 2). The same substitutions in the γ‐core region were applied before to create the γ‐core optimized variant of *P. chrysogenum* PAF, the PAF^opt^ (Sonderegger et al., 2018). In this protein, an already positively charged and hydrophilic γ‐core region was substituted with a more positive and hydrophilic one. PAF^opt^ proved to be more active against *C. albicans* (Sonderegger et al., 2018), and had a different antifungal spectrum against filamentous plant pathogenic fungi than that of the wild‐type PAF (Tóth et al., 2020). In contrast to NFAP2 (Figure 3a), this γ‐core modification of PAF did not influence dramatically the secondary structural elements of the protein, but PAF^opt^ showed significantly lower melting temperature and irreversible thermal unfolding (Sonderegger et al., 2018). After the γ‐core substitution, total structural disorganization of NFAP2 was observed (Figure 3a). All of these results indicate that the γ‐core modulation is not an efficient tool to improve the antifungal efficacy or to change the antifungal spectrum of NFAP2. However, disruption of the steric structure upon modification of the γ‐core suggests that this motif may affect the formation of the biologically active three‐dimensional structure of NFAP2.

It was demonstrated that a plasma membrane destruction mechanism is behind the antifungal activity of NFAP2 (Kovács et al., 2019; Tóth et al., 2018). Despite the different disulfide bonding patterns and disorganized structures, NFAP2*abbacc* and NFAP2γ were also able to destroy the plasma membrane integrity of *C. albicans* as the rNFAP2. This observation allows the deduction that this mechanism does not require an ordered protein structure, and it may not be connected to the γ‐core region; furthermore, it supports our previous hypothesis that the positively charged and highly hydrophilic unordered mid‐N‐terminal loop region is responsible for the plasma membrane destruction ability of NFAP2 on yeast cells (Tóth et al., 2018). However, further experiments focusing on fungal cell membrane‐NFAP2 interactions are needed to prove this assumption.

The emerging number of fungal infections drives the antifungal drug market with a 3.3% estimated compound annual growth rate during the forecast period, from USD 10.24 billion (2019) to USD 13.17 billion (2027). Nowadays, the first line of clinical therapy for fungal infections mainly includes azoles, polyenes, and echinocandins; however, as a consequence of an increasing number of resistant strains, the development of new antifungal drugs with limited potential for resistance development and better therapeutic outcome can further positively influence this market growth (Fortune Business Insights, n.d.). We hope that our present study can facilitate this aim as it allows the finalization of the solution structure determination of NFAP2, and contributes to the revelation of the designable structural elements of NFAP2 for future drug design.

## 4 MATERIALS AND METHODS

### 4.1 Proteins synthesis

Proteins were synthesized by stepwise solid‐phase peptide synthesis applying 9‐fluorenylmethyloxycarbonyl (Fmoc)/tert‐butyl (tBu) chemistry. To obtain a free carboxyl group at the C‐terminus, Fmoc‐Thr(tBu)‐OH, the appropriately protected C‐terminal amino acid was attached to PL‐Wang resin by DCC/HOBt coupling in the presence of 4‐dimethylaminopyridine catalyst first. Resin substitution was determined by the Gisin test. Proteins were prepared on the pre‐loaded resin using microwave‐assisted peptide synthesis and a Liberty Blue peptide synthesizer (CEM Corporation, Matthews, NC, USA) using Oxyma/DIC coupling. Proteins were cleaved off the resin with a trifluoroacetic acid (TFA)/water/dithiothreitol (DTT) (95%:5%:3% v/v:v/v:m/v) cocktail in 3 h. TFA was removed by evaporation, and the protein was precipitated with ice‐cold diethyl ether.

To achieve regioselective disulfide bond formation, orthogonal protecting groups were used for the sulfhydryl groups of cysteines. Trityl, the most commonly used protecting group in Fmoc/tBu chemistry, was applied for the first pair of cysteines. Trityl is removed together with the cleavage of the peptide from the resin. Side chains of the second pair were protected by acetamidomethyl (Acm), which was cleaved by 5 equiv. iodine in an 80% (V/V) aqueous solution of methanol containing 1 M of hydrochloric acid in 30 min. After the cleavage, the excess iodine was reduced by L‐ascorbic acid, and the reaction mixture was subjected to RP‐HPLC purification. Sulfhydryl groups of the third pair of cysteines were protected by 4‐methoxybenzyl (Mob), which was removed by a trifluoromethanesulfonic acid (TFMSA)/TFA/anisole (10%:80%:10% v/v:v/v:v/v) mixture at 0°C in 45 min. Typically, a 100 μL ice‐cold cleavage cocktail was added to 0.3 mg of protein, and the reaction was conducted in an ice bath. After 45 min 2.4 mL of ice‐cold distilled water was added to the mixture and it was extracted three times using 2.5 mL of diethyl ether to remove TFMSA and TFA. The aqueous phase was titrated with a 0.1 M solution of iodine in methanol until the permanent yellow color, and oxidation of sulfhydryl groups was performed by stirring the solution for 30 min. Then L‐ascorbic acid was added to the solution until decolorization and the mixture was purified by RP‐HPLC.

### 4.2 Recombinant NFAP2 production

rNFAP2 was produced by a *P. chrysogenum*‐based expression system in a minimal medium according to Tóth et al. (2018) and purified with a cation‐exchange chromatography, followed by an additional semipreparative RP‐HPLC step to reach ~100% purity (Kovács et al., 2019). For ^15^N‐/^13^C‐isotope labeling of NFAP2, the minimal medium was supplemented with Na^15^NO_3_ (0.3% w/v) and ^13^C glucose (1% w/v) (Cambridge Isotope Laboratories; Andover, MA, USA) as nitrogen and carbon sources, respectively.

### 4.3 RP‐HPLC analysis

Crude proteins were purified by semi‐preparative RP‐HPLC using a solvent system of (A) 0.1% TFA and (B) 80% acetonitrile (ACN), 0.1% TFA, and a linear gradient from 0% to 30% (B) in 60 min. Purification was performed either on a Phenomenex Jupiter Proteo 90 Å column (250 × 21.2 mm, Aschaffenburg, Germany) at a flow rate of 4 mL min^−1^ or Phenomenex Jupiter Proteo 90 Å column (250 × 10 mm) at a flow rate of 3 mL min^−1^ using a Shimadzu HPLC apparatus (Duisburg, Germany). Absorbance was detected at 220 nm. The purity was evaluated by analytical RP‐HPLC applying a linear gradient from 15% to 30% (B) in 15 min. Analytical RP‐HPLC and a linear gradient from 5% to 30% (B) in 25 min were used to follow the stepwise formation of disulfide bonds on a 4.6 × 250 mm Phenomenex Luna 10 μ C18 100A column and an Agilent 1100 HPLC (Palo Alto, CA, USA).

### 4.4 Mass spectrometry

Molecular mass measurements were performed on a Q Exactive Quadrupole‐Orbitrap mass spectrometer (Thermo Fisher Scientific, Waltham, MA, USA) equipped with a heated electrospray ion source with a flow injection method. Flow was provided by a Waters Acquity UPLC (Waters MS Technologies, Manchester, UK) system with flow rate: 100 μL min^−1^ eluent A: water with 0.1% (v/v) formic acid, eluent B: acetonitrile with 0.1% (v/v) formic acid; A and B eluent were mixed in 1:1 ratio. Multiply charged spectra of protein quasi‐molecular ions were deconvoluted using the deconvolution software included in the Xcalibur software (Thermo Fisher Scientific, Waltham, MA, USA).

### 4.5 Protein gel electrophoresis

Electrophoretic mobility and purity of the synthesized NFAP2 disulfide isomers, NFAP2γ, and rNFAP2 were investigated by 18% (w/v) tris–glycine SDS‐PAGE using Coomassie Brilliant Blue R‐250 staining.

### 4.6 Electronic circular dichroism spectroscopy

The secondary structure and folding integrity of rNFAP2, its disulfide isomers, and γ‐core variant were investigated using ECD spectroscopy. Spectra were acquired in the 195–260 nm wavelength range, at a scan speed of 100 nm s^−1^ using a Jasco‐J815 spectropolarimeter (JASCO Corporation, Tokyo, Japan). Protein samples were dissolved in ddH_2_O at 0.1 mg mL^−1^ concentrations. A 0.1 cm path‐length quartz cuvette was employed for all measurements. The measurement protocol consisted of the following steps. First, the samples were measured at 25°C. The temperature was then gradually increased up to 95°C at a rate of 5°C min^−1^ using a Peltier thermoelectric controller (TE Technology, Traverse City, MI, USA). During the heating of the samples, the ellipticity data at 228 nm was recorded as a function of temperature. This characteristic wavelength was determined previously for NFAP2 as well as other members of this protein family (Fizil et al., 2015; Galgóczy et al., 2017; Garrigues et al., 2017; Tóth et al., 2018). The samples were equilibrated for 1 min at each temperature point before measurements were taken. ECD spectra were recorded again in the 195–260 nm range at 95°C, the final temperature point of the unfolding experiment. The protein solutions were then left to cool to 25°C and additional ECD spectra were recorded in the 195–260 nm range after 5 min equilibration. The melting curves resulting from the single wavelength measurements in the 25–95°C temperature range were fitted with a symmetrical sigmoidal function. The melting point (*T*_*m*_) of protein structures was appointed by the *x*‐axis projections of the inflection points of the fitted curves. The reported spectra are accumulations of 10 scans, from which the corresponding solvent spectra were subtracted. Ellipticity data are presented in molar ellipticity (Θ_M_) units.

### 4.7 Nuclear magnetic resonance spectroscopy

The NMR experiments were carried out on Bruker Avance II (500 MHz) and NEO (700 MHz) spectrometers (Billerica, MA, USA) at 298 K temperature. NFAP2 samples were prepared in 20 mM acetate buffer adjusted to pH 4.5. 1.5 mg ^15^N‐labeled rNFAP2 and 0.57 mg unlabeled synthetic NFAP2*abbcac* were dissolved in 280 μL acetate buffer (H_2_O/D_2_O 95/5% mixtures) and measured in Shigemi NMR tubes to ensure higher sensitivity. For ^1^H‐^15^N HSQC spectra, the manufacturer's fhsqcf3gpph and sfhmqcf3gpph pulse sequences were used. The ^1^H‐^13^C HSQC spectra were recorded using the manufacturer's hsqcetgpprsisp2.2 and hsqcetgpsi2 pulse programs.

### 4.8 Antifungal susceptibility testing

A broth microdilution susceptibility testing method, according to Tóth et al. (2018) was applied to determine the MICs of rNFAP2, its synthetic disulfide isomers, and synthetic NFAP2γ for *C. albicans* CBS5982. The susceptibility test was performed in a low ionic strength broth medium (LCM: 0.5% glucose, 0.25% yeast extract, 0.0125% peptone (w/v)). Briefly, 100 μL of protein solution (25–100 μg mL^−1^ in twofold dilutions in LCM) was mixed with 100 μL of 2 × 10^5^ yeast cells mL^−1^ prepared in LCM in a flat‐bottom 96‐well microtiter plate (TC Plate 96 Well, Suspension, F; Sarstedt, Nümbrecht, Germany). LCM without NFAP2 was added to the cell suspension to serve as the untreated growth control. Two hundred microliters LCM was applied for the background calibration. The microtiter plates were incubated statically for 48 h at 30°C, and then the cells in the wells were mixed by pipetting. After that, the absorbance (OD_620_) of each well was measured with a microtiter plate reader (Multiscan Ascent, Thermo Scientific, Waltham, MA, USA). The absorbance of the untreated control represented 100% growth for the MIC calculation. MIC was defined as the lowest protein concentration at which growth was ≤5% in comparison with the untreated control. Susceptibility tests were repeated three times, including two technical replicates.

### 4.9 Fluorescence‐activated cell sorting analysis

Mid‐log phase *C. albicans* CBS 5982 cells (2 × 10^7^ cells mL^−1^) were exposed to MICs of rNFAP2, its synthetic disulfide isomers, or synthetic NFAP2γ for 16 h at 30°C under shaking at 160 rpm. After this incubation period, cells were collected by centrifugation (9000 × *g* for 5 min), washed with phosphate‐buffered saline (PBS) (pH 7.4), stained with 5 μg mL^−1^ PI (Sigma–Aldrich, St Louis, MO, USA) for 10 min at room temperature in the dark, and washed two times with PBS (pH 7.4), then they were resuspended in PBS (pH 7.4). Untreated and unstained cells were used as a negative control, untreated but PI‐stained cells as natural cell death control, and 50% (v/v) ethanol‐treated (10 min at 30°C under shaking at 160 rpm) cells as a positive control for PI staining. The proportion of PI‐positive (dead cells) in the samples was determined using a FlowSight imaging flow cytometer (Amins, Merck Millipore, Billerica, MA, USA). For data analysis, the Image Data Exploration and Analysis software (IDEAS; Amins, Millipore, Billerica, MA, USA) was applied. The PI‐positive portion of cells was determined by the gate excluding at least 99% of cells in the unstained control. One‐way ANOVA and Tukey's HSD post‐hoc tests were performed using the Statistics Kingdom online platform (https://www.statskingdom.com/index.html) to reveal significant differences between the proportion of the dead cells after the treatments with different NFAP2 disulfide isomers and γ‐core variants (Statistics Kingdom 2022). FACS analysis was repeated two times.

## AUTHOR CONTRIBUTIONS

Györgyi Váradi, Gábor Rákhely, Gábor K. Tóth, Gyula Batta, and László Galgóczy conceived and supervised the study, designed experiments, and edited the manuscript; Györgyi Váradi performed protein synthesis and RP‐HPLC chromatography; Zoltán Kele performed MS analyses; Attila Borics performed ECD spectroscopy; András Czajlik and Gyula Batta performed NMR spectroscopy; Gábor Bende performed protein gel electrophoresis and antifungal susceptibility tests, Csaba Papp performed FACS analyses; and Györgyi Váradi, Gábor K. Tóth, Gábor Rákhely, Gyula Batta, and László Galgóczy wrote the manuscript and revised it.

## Supporting information


**Figure S1.** Tryptic and chymotryptic sites in NFAP2 for disulfide linkage pattern determination with traditional mass spectrometry‐based method.
**Figure S2.** Visualization of the preliminary nuclear magnetic resonance structure of *Neosartorya* (*Aspergillus*) *fischeri* antifungal protein 2 and the predicted disulfide linkage patterns.
**Figure S3.** Mass spectrum of *Neosartorya* (*Aspergillus*) *fischeri* antifungal protein 2 (NFAP2) *abbacc* disulfide isomer.
**Figure S4.** Mass spectrum of *Neosartorya* (*Aspergillus*) *fischeri* antifungal protein 2 (NFAP2) *abbcac* disulfide isomer.
**Figure S5.** Mass spectrum of *Neosartorya* (*Aspergillus*) *fischeri* antifungal protein 2 (NFAP2) γ‐core variant. The attached .pdb file (NFAP2_preNMR.pdb) is the preliminary NMR structure of NFAP2.

NFAP2_preNMR is the preliminary NMR structure of NFAP2.
