# Supplementary material for: Hard nut to crack: Solving the disulfide linkage pattern of the Neosartorya (Aspergillus) fischeri antifungal protein 2
Source: Protein Sci. 2023 Jul 1;32(7):e4692. doi: 10.1002/pro.4692 (PMC10273333; doi:10.1002/pro.4692)
Supplement: Supplementary file 1 — Figure S1. Tryptic and chymotryptic sites in NFAP2 for disulfide linkage pattern determination with traditional mass spectrometry‐based method. Figure S2. Visualization of the preliminary nuclear magnetic resonance structure of Neosartorya (Aspergillus) fischeri antifungal protein 2 and the predicted disulfide linkage patterns. Figure S3. Mass spectrum of Neosartorya (Aspergillus) fischeri antifungal protein 2 (NFAP2) abbacc disulfide isomer. Figure S4. Mass spectrum of Neosartorya (Aspergillus) fischeri antifungal protein 2 (NFAP2) abbcac disulfide isomer. Figure S5. Mass spectrum of Neosartorya (Aspergillus) fischeri antifungal protein 2 (NFAP2) γ‐core variant. The attached .pdb file (NFAP2_preNMR.pdb) is the preliminary NMR structure of NFAP2. [file PRO-32-e4692-s001.docx]

**Hard nut to crack:** **Solving the disulfide linkage pattern of the *Neosartorya* (*Aspergillus*) *fischeri* antifungal protein 2**

Györgyi Váradi^1^, Zoltán Kele^1^, András Czajlik^2,3^, Attila, Borics^4^, Gábor Bende^5^, Csaba Papp^6^, Gábor Rákhely^5,7^, Gábor K. Tóth^1,8^, Gyula Batta^2^, László Galgóczy^5,9^

^1^Department of Medical Chemistry, Albert Szent-Györgyi Medical School, University of Szeged, Szeged, Hungary

^2^Department of Organic Chemistry, Faculty of Science and Technology, University of Debrecen, Debrecen, Hungary

^3^Department of Biochemistry, Institute of Biochemistry and Molecular Biology, Semmelweis University, Budapest, Hungary

^4^Laboratory of Chemical Biology, Institute of Biochemistry, Biological Research Centre, Eötvös Loránd Research Network, Szeged, Hungary

^5^Department of Biotechnology, Faculty of Science and Informatics, University of Szeged, Szeged, Hungary

^6^Department of Microbiology, Faculty of Science and Informatics, University of Szeged, Szeged, Hungary

^7^Institute of Biophysics, Biological Research Centre, Eötvös Loránd Research Network, Szeged, Hungary

^8^MTA-SZTE Biomimetic Systems Research Group, University of Szeged, Szeged, Hungary

^9^Fungal Genomics and Evolution Lab, Institute of Biochemistry, Biological Research Centre, Eötvös Loránd Research Network, Szeged, Hungary

**Correspondence**

Györgyi Váradi, Department of Medical Chemistry, Albert Szent-Györgyi Medical School, University of Szeged, Dóm tér 8, H-6720 Szeged, Hungary

TEL.: +36 62 545-142, FAX: +36 62 545-971

Email: varadi.gyorgyi@med.u-szeged.hu

László Galgóczy, Department of Biotechnology, Faculty of Science and Informatics, University of Szeged, Közép fasor 52, H-6726 Szeged, Hungary

TEL.: +36 62 546-936, FAX: +36 62 544-352

Email: galgoczi@bio.u-szeged.hu

**Running title:** Disulfide pattern of NFAP2

IATSPYY**ACNCPNNCK**HKKGSGCKYHSGPSDKSKVISGKCEWQGGQLNCIAT

**Supplementary Figure S1.** Tryptic and chymotryptic sites in NFAP2 for disulfide linkage pattern determination with a traditional mass spectrometry-based method. Blue and green letters indicate chymotryptic and tryptic cleavage sites, respectively. Red letters indicate cysteine residues. No cleavage sites exist within the **ACNCPNNCK** sequence.


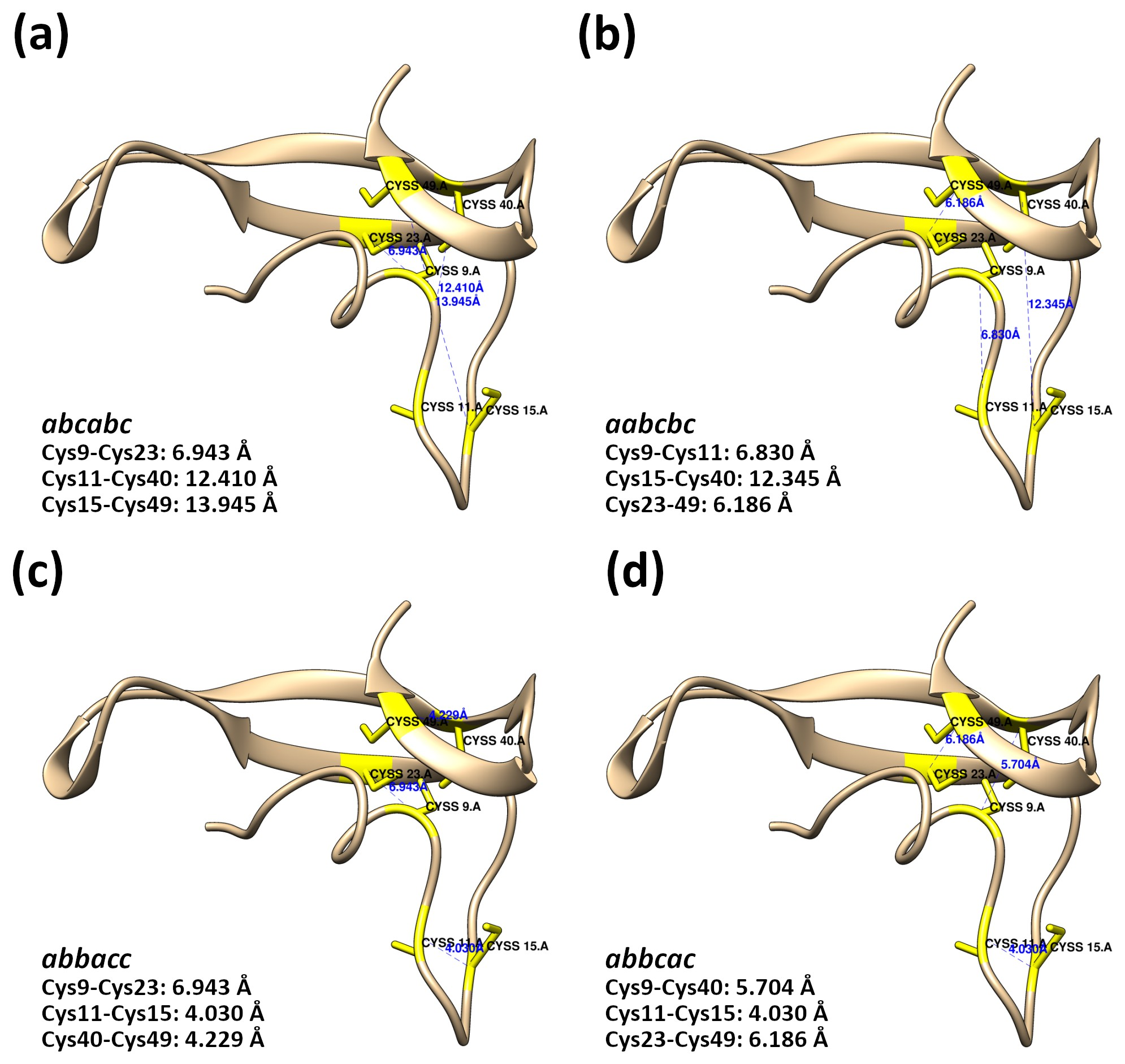


**Supplementary Figure S2.** Visualization of the preliminary nuclear magnetic resonance (NMR) structure of *Neosartorya* (*Aspergillus*) *fischeri* antifungal protein 2 (NFAP2) and the predicted disulfide linkage patterns. Disulfide linkage patterns were predicted from the primary structure with **(a)** DISULFIND,^1^ **(b)** DIANNA,^2^ **(c)** DisLocate;^3^ and from the preliminary NMR structure with **(d)** Disulfide by Design 2.0 servers.^4^ The tertiary structure of NFAP2 was visualized, and the distances between Cα atoms of cysteine pairs were measured with the use of UCSF Chimera software.^6^ Cysteine residues are indicated with yellow. According to a distance criterion between Cα atoms of a cysteine pair in the range of 3.0 Å and 7.5 Å,^6^ the *abbacc* and *abbcac* isomers are possible.


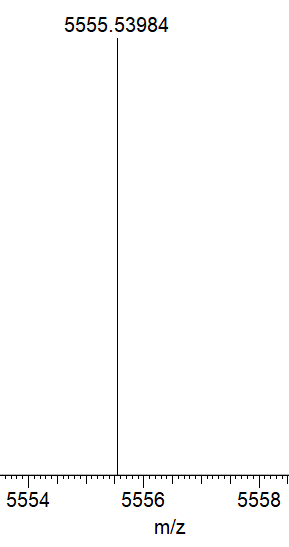

**Supplementary Figure S3.** Mass spectrum of *Neosartorya* (*Aspergillus*) *fischeri* antifungal protein 2 (NFAP2) *abbacc* disulfide isomer. The inset shows the deconvoluted spectrum of the protein (M+H^+^ monoisotopic).


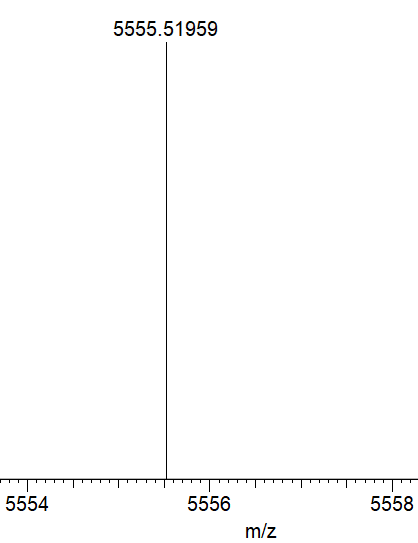


**Supplementary Figure S4.** Mass spectrum of *Neosartorya* (*Aspergillus*) *fischeri* antifungal protein 2 (NFAP2) *abbcac* disulfide isomer. The inset shows the deconvoluted spectrum of the protein (M+H^+^ monoisotopic).


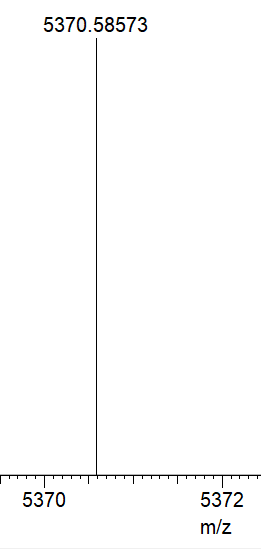


**Supplementary Figure S5.** Mass spectrum of *Neosartorya* (*Aspergillus*) *fischeri* antifungal protein 2 (NFAP2) γ-core variant. The inset shows the deconvoluted spectrum of the protein (M+H^+^ monoisotopic).

**References**

1. Ceroni A, Passerini A, Vullo A, Frasconi P (2006) DISULFIND: a disulfide bonding state and cysteine connectivity prediction server. Nucleic Acids Res 34:W177-181.

2. Ferrè F, Clote P (2006) DiANNA 1.1: an extension of the DiANNA web server for ternary cysteine classification. Nucleic Acids Res 34:W182-185.

3. Savojardo C, Fariselli P, Alhamdoosh M, Martelli PL, Pierleoni A, Casadio R (2011) Improving the prediction of disulfide bonds in Eukaryotes with machine learning methods and protein subcellular localization. Bioinformatics 27:2224-2230.

4. Craig DB, Dombkowski AA (2013) Disulfide by Design 2.0: a web-based tool for disulfide engineering in proteins. BMC Bioinformatics 14:346.

5. Pettersen EF, Goddard TD, Huang CC, Couch GS, Greenblatt DM, Meng EC, Ferrin TE (2004) UCSF Chimera--a visualization system for exploratory research and analysis. J Comput Chem 25:1605-1612.

6. Gao X, Dong X, Li X, Liu Z, Liu H (2020) Prediction of disulfide bond engineering sites using a machine learning method. Sci Rep 10:10330. Erratum in: Sci Rep. 2020; 10:12942.
